# RNA Sequencing of Formalin-Fixed, Paraffin-Embedded Specimens for Gene Expression Quantification and Data Mining

**DOI:** 10.1155/2016/9837310

**Published:** 2016-09-28

**Authors:** Yan Guo, Jie Wu, Shilin Zhao, Fei Ye, Yinghao Su, Travis Clark, Quanhu Sheng, Brian Lehmann, Xiao-ou Shu, Qiuyin Cai

**Affiliations:** ^1^Department of Cancer Biology, Vanderbilt University, Nashville, TN, USA; ^2^Division of Epidemiology, Department of Medicine, Vanderbilt Epidemiology Center and Vanderbilt-Ingram Cancer Center, Vanderbilt University Medical Center, Nashville, TN, USA; ^3^Department of Biostatistics, Vanderbilt University, Nashville, TN, USA; ^4^Genentech, Baltimore, MD, USA; ^5^Department of Biochemistry, Vanderbilt University, Nashville, TN, USA

## Abstract

*Background*. Proper rRNA depletion is crucial for the successful utilization of FFPE specimens when studying gene expression. We performed a study to evaluate two major rRNA depletion methods: Ribo-Zero and RNase H. RNAs extracted from 4 samples were treated with the two rRNA depletion methods in duplicate and sequenced (*N* = 16). We evaluated their reducibility, ability to detect RNA, and ability to molecularly subtype these triple negative breast cancer specimens.* Results*. Both rRNA depletion methods produced consistent data between the technical replicates. We found that the RNase H method produced higher quality RNAseq data as compared to the Ribo-Zero method. In addition, we evaluated the RNAseq data generated from the FFPE tissue samples for noncoding RNA, including lncRNA, enhancer/super enhancer RNA, and single nucleotide variation (SNV). We found that the RNase H is more suitable for detecting high-quality, noncoding RNAs as compared to the Ribo-Zero and provided more consistent molecular subtype identification between replicates. Unfortunately, neither method produced reliable SNV data.* Conclusions*. In conclusion, for FFPE specimens, the RNase H rRNA depletion method performed better than the Ribo-Zero. Neither method generates data sufficient for SNV detection.

## 1. Background

Formalin-fixed paraffin-embedded (FFPE) tissue is the most common method of tissue preparation used in clinics. FFPE preservation was developed to maintain morphology without any special considerations of preserving nucleic acids. Therefore, the difficulty of evaluating gene expression levels in FFPE samples remains one of the biggest disadvantages of FFPE preservation because the process of fixing the tissue samples and embedding them in paraffin often leads to RNA degradation and chemical modification. Furthermore, a nucleic acid can be cross-linked with a protein during the formalin fixation process, and most of the RNA isolated from FFPE tissues is highly degraded and reduced to a much lower yield than that of RNA isolated from the same amount of fresh tissues. To that end, RNA isolated from recently embedded tissues will be of better quality than RNA isolated from older embedded tissues. As a result, when amplifying RNA with oligo-dT primers, there is an overrepresentation of 3′ data due to the fragmented nature of RNA isolated from FFPE tissues.

Given the aforementioned reasons, gene expression analysis based on FFPE samples has been historically challenging. The most critical step in a FFPE sample based study is tissue preparation, as it ensures the integrity of the yield and data quality. It has been greatly emphasized that improper FFPE tissue preparation can diminish the quality of the nucleic acids from the tissue, limiting their use for gene expression profiling [1]. Yet, FFPE samples are often sought after due to their in-depth retrospective records. The success of a FFPE sample based study often depends on several steps: RNA isolation, reverse transcription, qPCR primer design, and preamplification. With carefully designed preparation protocols, FFPE samples have been proven to be an invaluable source for gene expression studies. The potential applications of FFPE samples in biomedical research are substantial.

The vast majority of cellular RNA (>80%) is composed of noninformative ribosomal RNAs (rRNAs, 28 S, 5.8 S, and 18 S rRNAs) that require removal prior to cDNA synthesis for a RNA-seq library. For high-quality RNA samples, polyadenylated RNA is enriched from intact RNA using oligo-dT primers. Since the rRNA does not have a poly-A tail, it is removed prior to cDNA synthesis along with other informative, non-polyA RNA species. RNA samples isolated from FFPE tissues have two features that are not compatible with oligo-dT primer selection: fragmented RNA that produces 3′ bias from oligo-dT selection, and, the degradation of the poly-A tail, thereby impacting the yield of recovered mRNA. Currently, there are two major rRNA depletion methods used for RNA isolated from FFPE samples: the Ribo-Zero rRNA removal kit (Epicentre/Illumina) and the RNase H method (also known as SDRNA) [2–4]. The Ribo-Zero kit uses a biotinylated antisense set of DNA capture probes that preferentially bind to rRNA. Magnetic beads are then used to capture the rRNA:DNA capture probe duplex. The resulting non-rRNA is left for cDNA synthesis. The RNase H method uses a similar initial depletion strategy by annealing 50–80 bp antisense DNA probes to the rRNA forming RNA:DNA hybrids. The RNA:DNA hybrids are treated with endoribonuclease RNase H that specifically degrades the phosphodiester bonds of RNA hybridized to DNA. This step is followed by a DNase I treatment to degrade the excess DNA probes. The resulting RNA is then ready for cDNA synthesis.

In the 2000s, microarray technology dominated high-throughput gene expression profiling but has since been replaced by RNAseq technology [5–9]. Successful gene expression studies based on FFPE samples by microarray technology [10–12] are much more abundant than studies using the relatively newer RNAseq technology. Here, we apply both RNA depletion methods, Ribo-Zero and RNase H, to isolated RNA from FFPE specimens to compare the overall qualities of data.

Furthermore, based on the premise that sequencing data offers exciting opportunities for additional data mining [13, 14, 16], we examined the data mining practicability of three types of supplementary information: SNVs, lncRNAs, and enhancer RNAs. SNVs are traditionally identified through DNA samples. SNV detection through RNAseq data has been historically challenging, although, with careful quality control, SNVs are detectable in RNAseq data [17–20]. Long noncoding RNAs (lncRNAs) are arbitrarily defined as longer than 200 nucleotides in length and do not encode proteins. Recent findings have suggested that lncRNAs play important roles in various diseases [21–28], and lncRNAs are detectable through the total RNAseq preparation method by the Ribo-Zero RNA rRNA removal kit [29]. Enhancer RNAs are a type of RNA that regulate spatiotemporal gene expression and impart cell-specific transcriptional outputs [30]. Recent advancements in RNAseq technology have enabled the ready detection of enhancer RNA [15, 30]. Super enhancer RNAs are a subset of enhancer RNA that are associated with cell identity and genetic risk of various diseases [31–33]. Our unique set of FFPE RNAseq data allows us to answer the question of whether a FFPE sample based RNAseq can be used for these types of data mining and determine which RNA isolation kit produces data most ideal for data mining.

## 2. Methods 

### 2.1. Sample Description

To evaluate the practicability and effectiveness of gene expression profiling using FFPE samples, we designed a study using four triple negative breast cancer (TNBC) FFPE tumor tissue samples. The H&E slides were reviewed by a study pathologist and tumor tissues were dissected from an unstained FFPE tissue section for total RNA extraction. The tumor tissue sections were stored in a vacuum chamber at 4°C for eight to nine years before RNA isolation was performed. Total RNA was extracted and purified using a Qiagen's miRNeasy FFPE Kit, a kit specifically designed for purifying the total RNA and microRNA from FFPE tissue sections. The input RNA amount for both Ribo-Zero and RNase H rRNA depletion methods was 200 ng each. The quantity and quality of the RNA samples extracted from tumor tissue FFPE sections were checked by Nanodrop (E260, E260/E280 ratio, spectrum 220–320 nm) and by separation on an Agilent BioAnalyzer. Total RNA extracted from each of the four tumors was split into two samples (for a total of eight samples). Two rRNA depletion methods were used: Ribo-Zero and RNase H. Each of the eight samples was treated with the two rRNA depletion methods, prepared for library using TruSeq RNA sample Prep Kit v2 (Illumina), and sequenced by BGI Americas. In total, 16 RNAseq libraries were generated following manufacture protocols and sequenced on two lanes (for a total of eight samples per lane). The qualified libraries were amplified on cBots to generate the cluster on the flow cell. The amplified flow cell was sequenced paired-end on the HiSeq 2000 at read length of the 90 base pairs.

### 2.2. Data Processing

RNAseq data was thoroughly quality-controlled at multiple stages (raw, alignment, and expression) following the recommendation by Guo et al. [34]. Raw data and alignment were quality-controlled using QC3 [35], while expression data was quality-controlled using MultiRankSeq [36]. Alignments were performed using Tophat 2 [37] against the HG19 human reference genome. Read counts for protein coding RNAs, lncRNAs enhancer RNAs, and super enchanter RNAs for each sample were obtained using HTSeq [38] against the collective General Transfer Format (GTF) file build from Ensembl Human GTF v74, Gencode lncRNA v 1.9, and enhancer RNA coordinates provided in [15]. Read count data for each type of the RNA was normalized to the total read counts of each sample. Cluster analysis was performed using Heatmap3 to identify similarities among samples [39]. Spearman's correlation coefficients were used to denote the distance between any two samples.

### 2.3. TNBC Subtype

Triple negative breast cancer (TNBC) is known to be molecularly and transcriptionally heterogeneous and can be classified into one of six subtypes (basal-like 1, BL1; basal-like 2, BL2; immunomodulatory, IM; mesenchymal, M; mesenchymal-stem like, MSL; and luminal AR, LAR) based on centroid correlations using gene expression [40]. In order to determine if RNAseq data originated from FFPE specimens can be used for clinical subtyping, we performed TNBC subtyping on each of the samples using TNBCtype [41] and compared the repeatability of TNBC subtyping consistency between the Ribo-Zero and RNase H methods.

### 2.4. NanoString

NanoString nCounter data was obtained on 302 genes using the same samples. The detailed processing and normalization method is described in [42]. We computed Spearman's correlation coefficients to evaluate the concordance between RNAseq and NanoString technology.

### 2.5. SNV Detection

We conducted advanced data mining on our FFPE RNAseq data to extract SNV. We inferred SNVs using Varscan 2 [43]. SNV quality was assessed by the transition/transversion (Ti/Tv) ratio and the pairwise heterozygous genotype consistency rate between any two samples. The Ti/Tv ratio is commonly used as a quality control measurement [44–46]. The Ti/Tv ratio of SNVs residing in coding regions should be between two and three and slightly lower for SNVs residing outside of the coding regions [47]. Higher Ti/Tv ratios, without exceeding the upper bound, usually indicate better overall quality. SNVs were annotated with ANNOVAR [48]. The heterozygous consistency rate of a pair of samples A-B is defined as the number of consistent genotypes between samples A and B, divided by the number of total heterozygous genotypes within B. A SNV is qualified as part of a consistency rate computation if it is detected by both samples and if the read depth for that SNV is at least 10 on both samples.

## 3. Results

### 3.1. Raw Data Quality Assessment

On average, the Ribo-Zero rRNA removal method produced 16.1 (range: 14.0–17.8) million reads per sample, and the RNase H produced 21.4 (range: 20.2–24.0) million reads per sample. The RNase H method consistently produced more reads than Ribo-Zero. Given that the same amount of RNA was used and the same number of samples was pooled per lane, a higher RNA capture efficiency is probable for RNase H than that of Ribo-Zero. On average, the guanine-cytosine (GC) content of Ribo-Zero was 72.3% (range: 70.0–76.8%), which was above the expected value (50%), whereas the GC content of the RNase H method was 50.6% (range: 39.8–55.1%). The GC content of the reference genome is roughly the expected GC content for the sequenced data. The GC content is 39.3% for the entire human genome, 48.9% for protein coding RNA, 39.7% for lncRNA, and 50.2% for rRNA. The sequenced reads of total RNAseq data are a mixture of protein coding RNA, lncRNA, and other species of RNA. With the expected GC content around 50%, RNase H produced data with GC content closest to the expected value. The raw data quality control only provided partial quality assessment of the samples.

### 3.2. Alignment Quality Assessment

Next, we examined the percentage of the reads that aligned to the coding region (Table 1). For the Ribo-Zero, on average, 34.7% (range: 27.4–46.1%) of the sequenced reads aligned to coding regions, and for the RNase H, on average, 71.6% (range: 42.6–80.3%) of the sequenced reads aligned to coding regions. An interesting observation was made in regard to the mapping quality (MQ). Ribo-Zero produced higher mapping quality data in the noncoding region, whereas the RNase H method produced higher mapping quality in the coding region. For the Ribo-Zero, the average MQ for the coding region was 29 (range: 23–36) and 47 (range: 46-47) for the noncoding region. For the RNase H method, the average MQ was 45 (range: 44−46) for the coding region and 34 (range: 33–41) for the noncoding region. One of the repeats of sample two, which used the RNase H, is a potential outlier because it had the lowest GC content (39.8%) and the lowest coding region alignment rate (42.6%) of all the RNase H based samples. RNase H also produced less percentage of rRNA reads compared to Ribo-Zero (paired *t*-test *p* = 0.03).

### 3.3. Cluster Analysis

**Figure 1:**
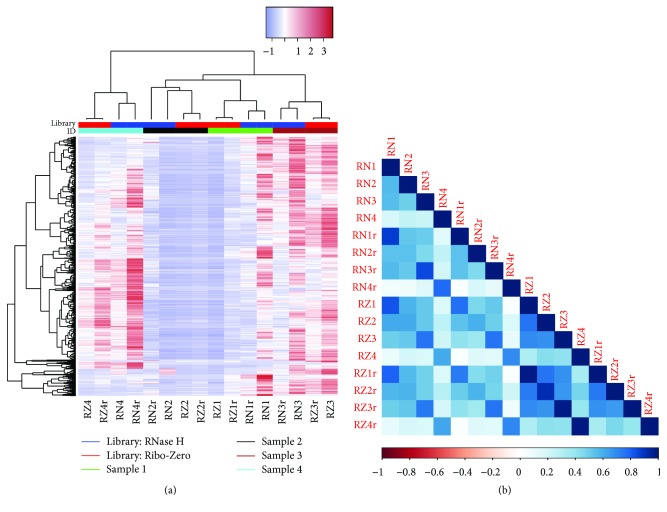
(a) Unsupervised cluster using all detected RNAs. Samples were clustered first by replicates then by rRNA depletion method. (b) Pairwise Spearman correlation heatmap between all samples. Ribo-Zero produced higher correlation between repeats than RNase H. The samples RN4 and RN4r produce low correlations with other samples compared to other random pairs. This could be the result of variation in the sample or variation introduced by the RNase H kit.

Cluster analysis showed that, regardless of which RNA isolation kit was used, the repeated sample clustered together based on gene expression. Within repeated samples, the rRNA depletion kits were clustered separately. The cluster analysis results provided additional evidence of quality concern for the RNase H sample two repeat one, as it was the only sample that did not perfectly cluster with its pair within the same RNA isolation kit (Figure 1(a)). The correlation heatmap (Figure 1(b)) showed similar results as presented in Figure 1(a). Essentially, we observed a higher pairwise correlation between repeated samples than between random samples.

### 3.4. TNBC Subtype Comparison

**Figure 2:**
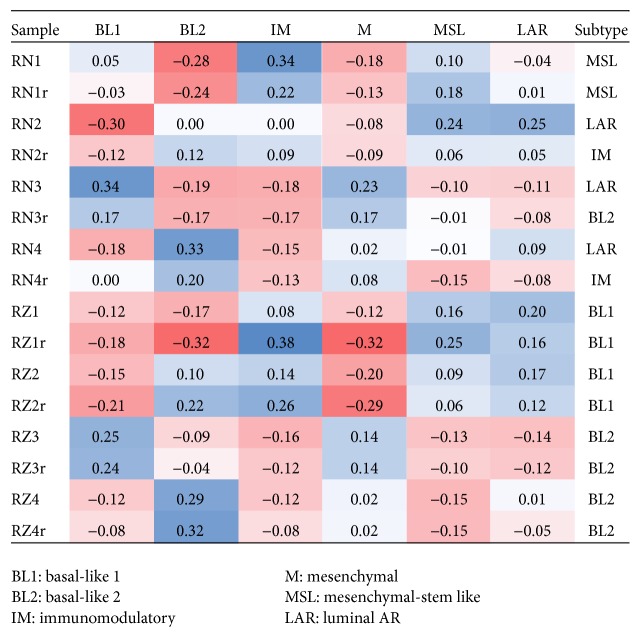
TNBC subtype results from TNBC type. The results show that RNase H samples produced better TNBC subtype consistency than Ribo-Zero samples.

Overall, correlations to the TNBC subtypes were similar in replicates (Figure 2). RNase H samples had more consistent TNBC subtype calls between replicates (3/4 matching) than the Ribo-Zero samples (2/4 matching). The nonmatching replicate in the RNase H samples is sample 2 where we have previously noted its quality issue. This result suggests that RNase H produces RNAseq data with more consistent TNBC subtyping.

### 3.5. NanoString Comparison

**Figure 3:**
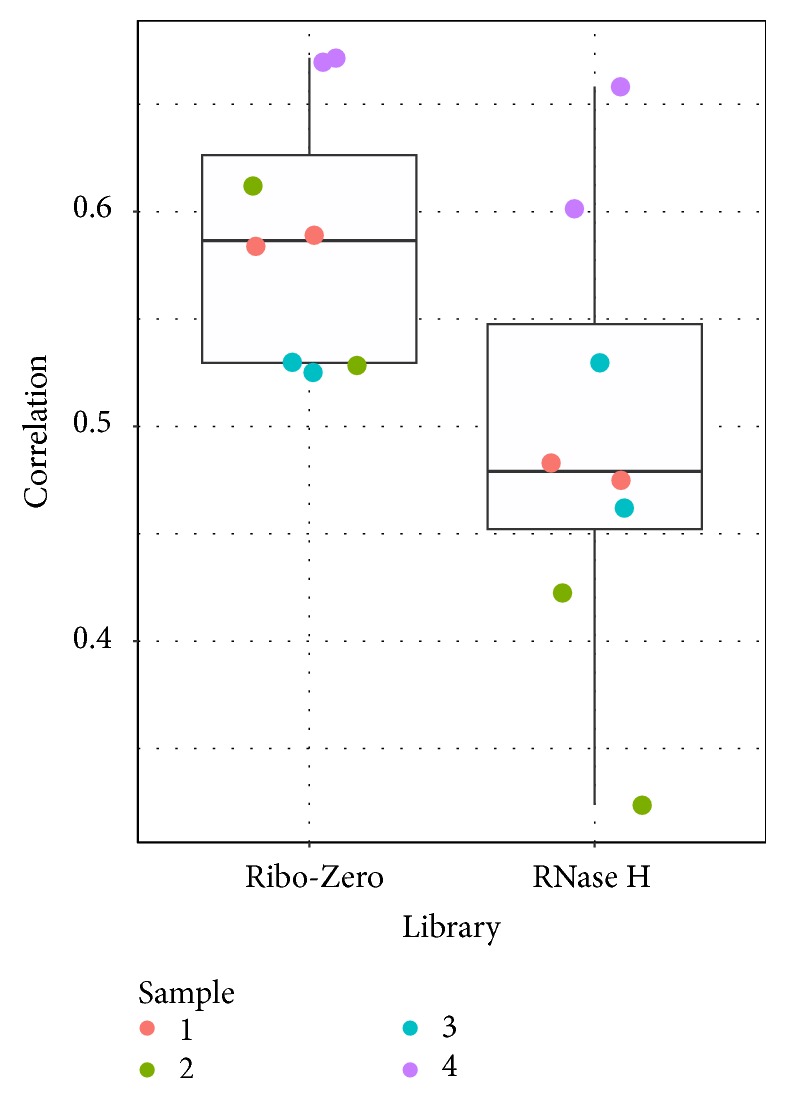
Spearman's correlation coefficients between RNAseq data and NanoString data. The Ribo-Zero samples produced slightly higher correlation with NanoString data then RNase H samples.

We computed Spearman's correlation coefficients using the gene expression levels between RNAseq. The correlation dot plot (Figure 3) shows that the average correlation between Ribo-Zero and NanoString is 0.59 (range: 0.53–0.67), and the average correlation between RNase H and NanoString is 0.49 (range: 0.32–0.66). The lowest correlation was produced by RNase H sample two repeat one which is likely to be a sample with a sequencing quality issue.

### 3.6. RNA Detection

**Figure 4:**
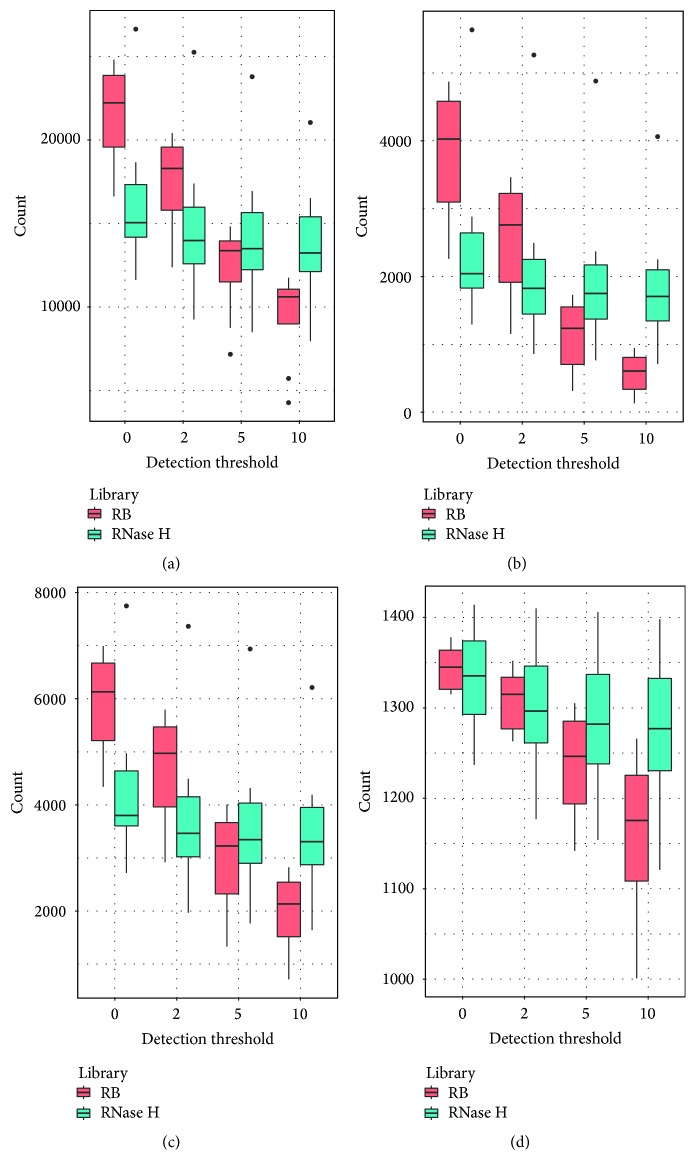
Detected RNA using thresholds: normalized reads count > 0, 2, 5, and 10. (a) Protein coding RNA. (b) lncRNA. (c) Enhancer RNA. (d) Super enhancer RNA. At lower thresholds (more noise), Ribo-Zero samples detected more RNAs. At higher thresholds (more reliability), RNase H method detected more RNAs.

We examined four kinds of RNAs: mRNA (Figure 4(a)), lncRNA (Figure 4(b)), enhancer RNA (Figure 4(c)), and super enhancer RNA (Figure 4(d)). After normalization by total read count, we used four detection thresholds (>0, >2, >5, and >10) to compare the RNA detection rates between the two RNA isolation kits. For all four types of RNAs, the Ribo-Zero rRNA depletion method detects more RNA at detection thresholds >0 and >2. When higher detection thresholds were used, the RNase H managed to detect more RNAs. RNA detected with low expression values could be the result of noises and is therefore less trustworthy than RNA detected with higher levels of expression. Based on these results, the RNase H rRNA depletion method detected more potentially reliable RNA as compared to the Ribo-Zero.

### 3.7. SNV Detection

**Figure 5:**
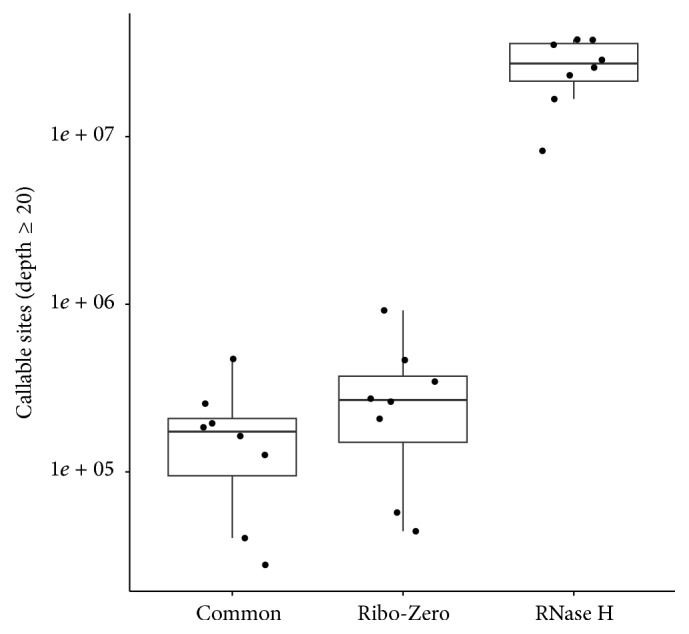
Callable site is defined as a genomic position with depth coverage ≥ 20. The number of callable sites indicates the number of genomic positions that are suitable for SNV inference. RNase H had substantially more callable sites than Ribo-Zero. The percentage of difference in callable site is significantly more than the percentage of difference in number of total reads sequenced by the two kits. *Y*-axis is plotted in log_10_ scale.

We inferred SNVs from the FFPE RNA data using VarScan 2. After filtering for high quality SNVs (depth > 20), on average, the Ribo-Zero samples identified 525 SNVs per sample (range: 73–1862), and the RNase H samples identified 57747 SNVs per sample (range: 21932–87146). The RNase H samples clearly identified more SNVs than the Ribo-Zero prepared samples. This is caused by the difference of number of callable sites between the two kits. We defined a callable site to be a genomic position with coverage depth ≥ 20. RNase H produced substantially more callable sites than Ribo-Zero (Figure 5). The callable site analysis result shows that the coverage of Ribo-Zero is more spread out than RNase H. High variations in the number of SNVs were observed for both RNA isolation kits. For SNVs identified in coding regions, on average, the Ti/Tv ratio for Ribo-Zero was 3.51 (range: 2.42–8.00) and 2.08 (range: 1.34–2.47) for RNase H. For SNVs identified in noncoding regions, on average, the Ti/Tv ratio for Ribo-Zero was 2.84 (range: 2.37–3.84) and 3.74 (range: 1.04–5.27) for RNase H. The variation for the Ti/Tv ratio is large, indicating potential problems with the SNVs identified.

Additional evidence for problematic SNV inferences was observed in the results of the pairwise heterozygous genotype consistency between samples. In DNA sequencing, we expect the heterozygous genotype consistency rate for technical replicates to be above 0.99. For RNAseq, the consistency rate is expected to be lower but still yield above 80%. However, on average, the consistency rates for both kits were less than 40% which were substantially below expectation. Restricting SNV pairwise heterozygous consistency computation to SNVs with depth greater than 50x for both samples in the pair increased the consistency slightly but still remained <50%. The low heterozygous consistency rates indicate that SNVs inferred from FFPE RNAseq samples contain high false positive rates and are therefore not ideal sources for detecting SNV.

## 4. Discussion

Utilization of FFPE specimens for gene expression studies could open a new avenue for molecular epidemiological and clinical research. Yet to date, the low quality of RNA from FFPE specimens for gene expression analysis has been a challenge. Several technologies have been developed for quantifying gene expression from FFPE specimens, such as NanoString [49] and quantitative Nuclease Protection Assay [50].

Since gene expression data can yield both molecular subtype classification and predicative markers of risk, efforts have been made to use RNA extracted from FFPE tissue on NanoString and microarray platforms [51, 52]. Triple negative breast cancer has been shown to be transcriptionally heterogeneous, with several molecular subtypes with differing biology [40, 53, 54]. The ability to identify TNBC subtypes from RNA isolated from FFPE tissues will provide opportunities for future clinical trial designs and retrospective evaluations of previously failed clinical trials by individual subtypes. To determine if RNA extracted from FFPE tissue that has been stored for eight to nine years could yield gene expression profiles by RNAseq sufficient enough to subtype TNBC, we compared the efficiencies of both the Ribo-Zero and the RNase H methods for rRNA depletion.

Through thorough quality control and analyses, we found that expression profiling of coding and noncoding RNA is possible for aged FFPE samples with RNAseq technology. The Ribo-Zero and RNase H method each had strengths and weaknesses in different areas. Our analyses suggested that RNase H is more suitable for studies that target protein coding RNA. On the other hand, Ribo-Zero offered more consistency between repeated samples, which is of pivotal importance, especially for low quality RNA extracted from FFPE tissues. Under the same amount of library input and same multiplexing scenario, RNase H consistently produced more reads than Ribo-Zero. Many reasons could have caused this read counts difference, including batch effect of the cluster on the flow cell, and library efficiencies. The evidences of more total reads sequenced under the same input amount and better rRNA depletion efficiency for RNase H support that RNase H has better library efficiency than Ribo-Zero. RNase H hybridizes directly to the sequences of rRNAs without the requirement of perfect match. The Ribo-Zero uses bait strategy which is similar to enrichment like exome capture with baits and beads. Thus it does not remove degraded, fragmented rRNAs as efficient as RNase H. Our study confirms previous finding that RNase H performed better than Ribo-Zero for low quality RNAs [55].

Furthermore, genes quantified from Ribo-Zero processed RNAseq data also had a slightly higher correlation with genes quantified by NanoString technology. This suggests that Ribo-Zero might offer better repeatability, although the correlation (50–60%, FFPE) with NanoString data (FFPE) did not reach the high correlation (80–90%, fresh frozen) between microarray and RNAseq [56]. We suspect this is primarily due to the variation introduced by the degraded quality of the RNA extracted from FFPE samples.

The subtyping of gene expression profiles obtained by both methods demonstrated that RNA isolated from stored FFPE samples can be used to determine distinct TNBC subtypes. While TNBC subtypes were similar among replicates, RNase H samples had more consistent TNBC subtype calls between replicates than that of the Ribo-Zero samples, which is potentially due to the more efficient capture of protein coding RNA.

By performing SNV detection analysis, we found that SNV detected by FFPE RNAseq data is subjected to quality concerns. It has been suggested that the SNV data inferred from RNAseq data has a high false positive rate [57]. Several factors can contribute to the high false positive rate of SNV. First, alignment on RNAseq data can be more complicated than DNA sequencing data [19]. Processes such as RNA editing, alternative splicing, gene fusion, and polyadenylation introduce additional complications in RNAseq alignment. The step that reverse-transcribes RNA to cDNA can also introduce random errors. We have found that the number of SNVs inferred from RNAseq data can be several folds higher than that from the exome sequencing data on the sample. In our study, the lower quality of RNA isolated from FFPE tissue will result in an even higher number of false positive SNVs. The low consistency rate of SNVs identified between paired samples suggests that RNAseq data from FFPE tissues are not suitable for SNV inference.

## 5. Conclusion

Recent studies have shown remarkably high consistency between RNAseq data generated from paired freshly frozen and FFPE tissue samples [58–60]. Our study provides additional evidence for the practicability of conducting gene expression RNAseq with FFPE tissues. There is no denying that there are technical and quality limitations for FFPE RNAseq data. However, the majority of the issues can be overcome through thorough quality control and careful bioinformatics analyses. Our study supports the notions that RNAseq on FFPE samples can be used as an unbiased and comprehensive assessment of gene expression in biomedical studies, and RNase H method provides more efficient rRNA depletion than Ribo-Zero method for low quality fragmented RNAs.

## Figures and Tables

**Table 1:** Sample description and alignment statistics.

ID	Library	Raw data			Alignment			
		Total reads	BQ	GC	CR	Non-CR	CR MQ	Non-CR MQ
1	Ribo-Zero	17.8 M	31	71.4%	27.7%	72.3%	32	47
2	Ribo-Zero	16.1 M	30	76.8%	31.4%	68.6%	23	47
3	Ribo-Zero	16.8 M	31	70.7%	31.6%	68.4%	28	46
4	Ribo-Zero	14.0 M	31	72.3%	46.0%	54.0%	34	47
1	Ribo-Zero	16.1 M	31	70.4%	27.4%	72.6%	31	47
2	Ribo-Zero	14.9 M	31	75.4%	37.9%	62.1%	21	47
3	Ribo-Zero	17.6 M	31	71.1%	29.8%	70.2%	29	47
4	Ribo-Zero	15.6 M	31	70.0%	46.1%	53.9%	36	47
1	RNase H	20.2 M	35	51.6%	79.9%	20.1%	46	33
2	RNase H	20.5 M	36	39.8%	42.6%	57.4%	45	41
3	RNase H	20.4 M	35	51.6%	78.9%	21.1%	45	33
4	RNase H	21.4 M	35	48.6%	58.0%	42.0%	45	37
1	RNase H	22.1 M	35	52.5%	80.3%	19.7%	46	35
2	RNase H	22.4 M	34	55.1%	74.7%	25.3%	44	33
3	RNase H	20.6 M	35	52.0%	78.5%	21.5%	45	31
4	RNase H	24.0 M	34	53.6%	80.1%	19.9%	45	30

CR: coding region; BQ: base quality; MQ: mapping quality; GC: GC content.

## Abbreviations

FFPE: Formalin-fixed paraffin-embedded
RNAseq: RNA sequencing
SNV: Single nucleotide variant
TNBC: Triple negative breast cancer.

## Additional Points

*Availability of Data and Materials.* The sequencing data used in this study have been deposited into Gene Expression Omnibus (GEO) under the accession number GSE74270.
